# Integration analysis of long non-coding RNA (lncRNA) role in tumorigenesis of colon adenocarcinoma

**DOI:** 10.1186/s12920-020-00757-2

**Published:** 2020-07-29

**Authors:** Arash Poursheikhani, Mohammad Reza Abbaszadegan, Negin Nokhandani, Mohammad Amin Kerachian

**Affiliations:** 1 Medical Genetics Research Center, Mashhad University of Medical Sciences, Mashhad, Iran; 2 Department of Medical Genetics, Faculty of Medicine, Mashhad University of Medical Sciences, Mashhad, Iran; 3 Immunology Research Center, Mashhad University of Medical Sciences, Mashhad, Iran; 4 Department of Immunology, School of Medicine, University of Golestan Medical Sciences, Gorgan, Iran; 5 Cancer Genetics Research Unit, Reza Radiotherapy and Oncology Center, Mashhad, Iran

**Keywords:** Colorectal cancer, Tumorigenesis, Long non-coding RNAs, MicroRNA

## Abstract

**Background:**

Colon adenocarcinoma (COAD) is one of the most common gastrointestinal cancers globally. Molecular aberrations of tumor suppressors and/or oncogenes are the main contributors to tumorigenesis. However, the exact underlying mechanisms of COAD pathogenesis are clearly not known yet. In this regard, there is an urgent need to indicate promising potential diagnostic and prognostic biomarkers in COAD patients.

**Methods:**

In the current study, level 3 RNA-Seq and miR-Seq data and corresponding clinical data of colon adenocarcinoma (COAD) were retrieved from the TCGA database. The “limma” package in R software was utilized to indicate the differentially expressed genes. For in silico functional analysis, GO and KEGG signaling pathways were conducted. PPI network was constructed based on the STRING online database by Cytoscape 3.7.2. A ceRNA network was also constructed by “GDCRNATools” package in R software. Kaplan-Meier survival analysis (log-rank test) and ROC curve analysis were used to indicate the diagnostic and prognostic values of the biomarkers.

**Results:**

The differential expression data demonstrated that 2995 mRNAs, 205 lncRNAs, and 345 miRNAs were differentially expressed in COAD. The GO and KEGG pathway analysis indicated that the differentially expressed mRNAs were primarily enriched in canonical processes in cancer. The PPI network showed that the CDKN2A, CCND1, MYC, E2F, CDK4, BRCA2, CDC25B, and CDKN1A proteins were the critical hubs. In addition, the Kaplan-Meier analysis revealed that 215 mRNAs, 14 lncRNAs, and 39 miRNAs were associated with overall survival time in the patients. Also, the ceRNA network data demonstrated that three lncRNAs including *MIR17HG, H19, SNHG1, KCNQ1OT1, MALAT1, GAS5, SNHG20, OR2A1-AS1,* and *MAGI2-AS3* genes were involved in the development of COAD.

**Conclusions:**

Our data suggested several promising lncRNAs in the diagnosis and prognosis of patients with COAD.

## Background

Colon adenocarcinoma (COAD) is one of the most common gastrointestinal (GI) cancers and is the second leading cause of cancer-related death, globally [1, 2]. It is demonstrated that COAD occurs in approximately 5% of overall population at any given time in the world [3]. Despite the current screenings and therapies such as endoscopic resection and radical surgery, nearly half of the patients are diagnosed as advanced cases of COAD, experiencing tumor recurrence and relapse. COAD tumorigenesis has complicated multi-step processes including colon epithelial cell proliferation, aberration in differentiation, apoptosis resistance, survival, and invasion mechanisms [4]. Molecular aberrations of tumor suppressors and/or oncogenes are also one of the main contributors in different types of tumors especially COAD tumorigenesis [5]. However, due to complicacy of the underlying molecular pathways, the exact pathogenic contributors of COAD have not yet been clarified. Hence, there is an urgent need to indicate promising diagnostic and prognostic biomarkers for COAD. Recent investigations have highlighted the role of non-coding RNAs in the tumorigenesis of various malignancies. Among different kinds of non-coding RNAs, long non-coding RNA (lncRNA) is a putative class of non-coding RNA with more than 200 nucleotides in length, without any open-reading-frame (ORF) to encode proteins [5, 6]. Interestingly, a large body of evidence indicates that lncRNAs plays critical roles in a variety of biological processes including cell proliferation, cellular development, differentiation, carcinogenesis, and metastasis through modulating gene expression at the transcriptional and posttranscriptional levels directly or by recruiting chromatin remodeling factors [6–8]. Aberrant expression of lncRNAs has been well-documented in different sorts of cancers [9]. Dysregulation of lncRNA *HOTAIR, H19, MALAT1, SNHG7, GAS8-AS,* and *NEAT1* were extensively well-studied and have been demonstrated to contribute in tumorigenesis and poor prognosis [5, 9–13]. Numerous investigations have shown that the lncRNAs can exert their function by competing endogenous RNA (ceRNA) crosstalk. For instance, it has been shown that lncRNA *SCARNA2* was overexpressed in COAD tissues and it remarkably correlated with chemoresistance. Mechanistically, *SCARNA2* via targeting *miR-342-3p*, upregulates *EGFR* and *BCL2* expression in COAD cells [14]. Furthermore, overexpression of lncRNA *SNHG1* has been shown to promote epithelial-mesenchymal transition (EMT) by binding to *miR-497/miR-195-5p* in COAD cells [15]. Also, lncRNA *BDNF-AS* was downregulated in COAD patients and served as a tumor suppressor gene. Unsurprisingly, ectopic expression of *BDNF-AS* suppressed cell proliferation and migration via epigenetically downregulating *GSK-3β* expression through *EZH2* [16]. Moreover, several investigations have considered lncRNAs as therapeutic opportunities in COAD. For instance, it has been demonstrated that overexpression of LINC00152 can promote Fascin actin-bundling protein 1 (FSCN1) expression via sponging miR-632 and miR-185-3p, which consequently leads to proliferation and metastasis in COAD [17]. A recent study has demonstrated that targeting lncRNA FLANC by 1,2-dioleoyl-sn-glycero-3-phosphatidylcholine nanoparticles loaded with a specific small interfering RNA, decreased metastasis without any significant toxicity. They proposed that FLANC may act as a novel therapeutic strategy in COAD [18].

Additionally, many researches have suggested the potency of lncRNAs as biomarkers in the blood and serum. They suggested microvesicles and exosomes as carriers, being protected and stabilized in circulation [19]. In the current study, we comprehensively investigate lncRNAs, miRNAs, and mRNAs expressions from a public database, “Cancer Genome Atlas (TCGA)” and we constructed a ceRNA network in COAD. Also, we proposed novel potential biomarkers for COAD.

## Methods

### Sample and data collection

Clinical data of COAD were retrieved from the TCGA database (https://portal.gdc.cancer.gov/repository). The inclusion criteria were: (1) the histopathological diagnosis was COAD; (2) having complete demographic data including age, vital status, race, ethnicity, pathological stage, TNM classification, and overall survival time. Totally, 459 COAD were enrolled in this study. Two hundred and thirty participants had age > 68 years and 229 patients had age ≤ 68 and 243 and 216 patients were male and female, respectively. Among 459 patients, only 4 patients were Hispanic or Latino and 271 were non-Hispanic or non-Latino. Two hundred and fourteen patients were white, 29 patients were Black or African American, 11 were Asian and 1 American Indian/Alaska native. Pathological stages of I, II, III, and IV were 76, 178, 129 and 65, respectively. The clinical characteristics are summarized in Table 1.

**Table 1:** Clinicopathological characteristics of COAD patients

**Characteristics**	**N**	**(%)**
**Age** (year) (mean ± SD)	66.92 (13)	
Age > 68	230	50.1
Age ≤ 68	229	49.9
**Sex**		
Male	243	52.9
Female	216	47.1
**Ethnicity**		
Hispanic or Latino	4	0.9
Not Hispanic or Latino	271	59
NA	184	40.1
**Race**		
American Indian or Alaska Native	1	0.2
Asian	11	2.4
Black or African American	59	12.9
White	214	46.6
NA	174	37.9
**Vital status**		
Alive	357	77.8
Dead	102	22.2
**Pathologic (stage)**		
Stage I	76	16.5
Stage II	178	38.7
Stage III	129	28.1
Stage IV	65	14.1
**Pathologic (T)**		
T1	11	2.4
T2	78	17
T3	313	68.2
T4	56	12.2
Tis	1	0.2
**Pathologic (M)**		
M0	337	73.4
M1	65	14.2
MX	50	10.9
NA	7	1.5
**Pathologic (N)**		
N0	270	58.8
N1	106	23.1
N2	83	18

*NA* Not Applicable

### RNA-Seq and miR-Seq data analysis

RNA-Seq and miR-Seq Level 3 data were collected from the TCGA database. The raw count of the reads of RNA-Seq and miR-Seq data was normalized by Voom and TMM normalization methods. All the analyses were conducted in R software. The “limma” package in R software was utilized to indicate the differentially expressed mRNAs (DEmRNAs), lncRNAs (DElncRNAs), and miRNAs (DEmiRNAs) between normal solid tissues and primary tumors. The concluded data were filtered based on the |log2 fold change (FC)| > 1 for DEmRNA, DElncRNA, and DEmiRNA. *P*-value < 0.05 and false discovery rate (FDR) < 0.05 were considered as significant thresholds.

### Functional enrichment analysis and protein-protein interaction (PPI) network

For in silico functional enrichment analysis, gene ontology (GO) in three domains including biological processes, cellular components, and molecular functions, in addition to Kyoto Encyclopedia of Genes and Genomes (KEGG) signaling pathways were conducted. The GO and KEGG outputs were visualized by R software (ggplot2 package). The PPI network was constructed based on the STRING online database by Cytoscape 3.7.2. Molecular Complex Detection (MCODE) was used to analyze and predict the interactions (score value > 0.4).

### LncRNA-miRNA-mRNA ceRNA network construction

LncRNA-miRNA-mRNA ceRNA network was constructed by “GDCRNATools” (http://bioconductor.org/packages/devel/ bioc/html/GDCRNATools.html) package in R software based on starbase database [14]. The nodes and edges were virtualized by Cytoscape 3.7.2.

### Statistical analysis

All the differentially expressed data were analyzed by using R software (3.5.2) through the “GDCRNATools” package. Kaplan-Meier survival analysis (log-rank test) was used to indicate the relation between over or downregulation of RNA, based on median expression with patient’s survival time. ROC curve analysis, univariate, and multivariate Cox regression analysis were conducted by SPSS v21. *P-value* < 0.05 was considered as a significant threshold.

## Results

### Differentially expressed genes

**Fig. 1:**
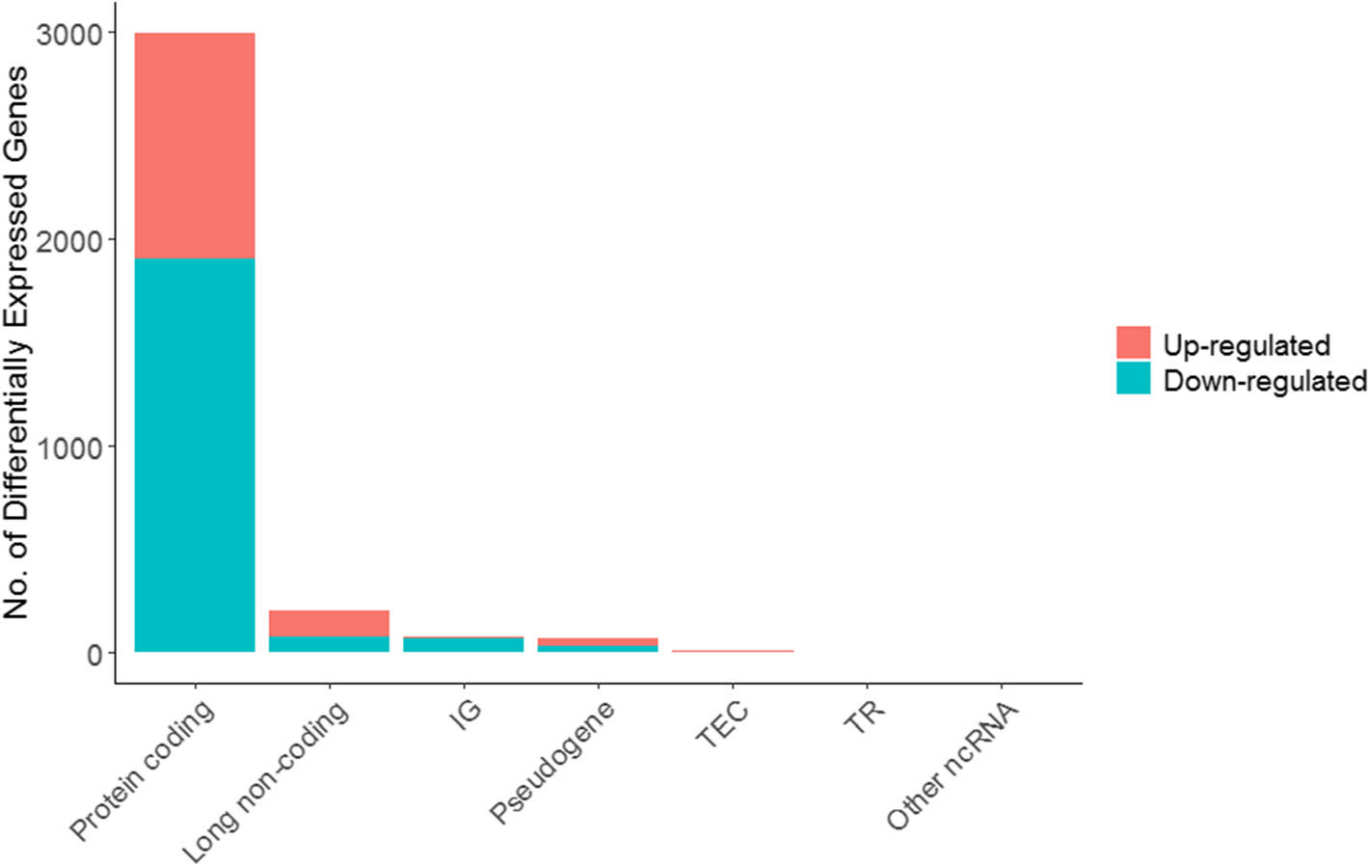
Bar graph of the differentially expressed genes in the COAD samples. TEC: To be Experimentally Confirmed; TR: T cell receptor; IG: Immunoglobulin

**Fig. 2:**
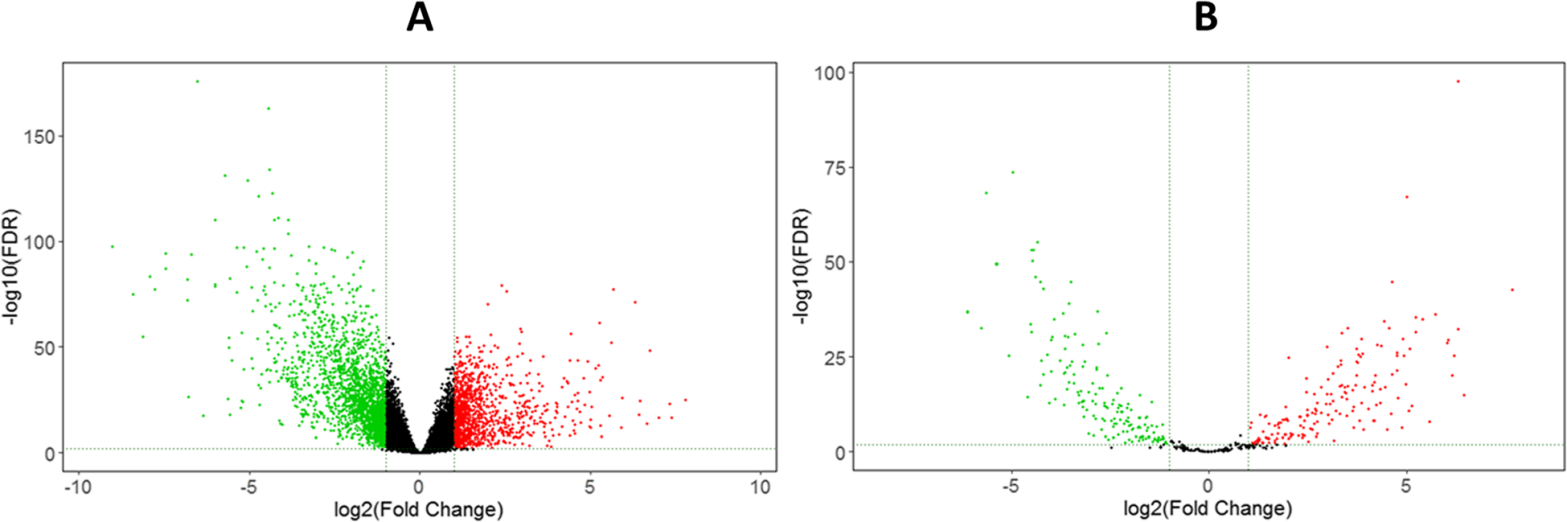
Volcano plot of the differentially expressed genes and miRNAs. **a** Volcano plot of differentially expressed lncRNAs and mRNAs. Overexpressed genes are demonstrated in red and down-regulated genes are demonstrated in green. **b** Volcano plot of differentially expressed miRNA. Overexpressed and down-regulated genes are demonstrated in red and green, respectively

Our data demonstrated that 2995 mRNAs (1094 up-regulated and 1901 down-regulated) were differentially expressed in COAD. Moreover, 205 lncRNAs (128 up-regulated and 77 down-regulated) were identified that were deferentially expressed in patients. Three hundred and forty-five miRNAs containing 183 up-regulated and 162 down-regulated have been found with differential expression in the COAD samples. The data are presented in Figs. 1, 2 and Tables 2, 3.

**Table 2:** Top 20 upregulated mRNAs, lncRNAs, and miRNAs

	**symbol**	**logFC**	**AveExpr**	**t**	***P*Value**	**FDR**	**B**
**mRNA**							
ENSG00000167755	KLK6	7.79	2.04	11.38	0.00	0.00	50.14
ENSG00000170373	CST1	7.34	2.57	10.84	0.00	0.00	45.36
ENSG00000137673	MMP7	7.02	4.16	9.01	0.00	0.00	30.34
ENSG00000167767	KRT80	6.75	4.50	16.83	0.00	0.00	104.28
ENSG00000185269	NOTUM	6.67	2.82	8.13	0.00	0.00	23.79
ENSG00000123500	COL10A1	6.43	2.48	9.40	0.00	0.00	33.40
ENSG00000062038	CDH3	6.33	5.96	21.72	0.00	0.00	157.90
ENSG00000164379	FOXQ1	5.94	4.48	11.52	0.00	0.00	51.46
ENSG00000165376	CLDN2	5.90	5.63	7.41	0.00	0.00	18.76
ENSG00000164283	ESM 1	5.68	2.10	23.05	0.00	0.00	172.24
ENSG00000105989	WNT2	5.64	2.35	17.66	0.00	0.00	113.19
ENSG00000060718	COL11A1	5.57	3.74	9.20	0.00	0.00	31.81
ENSG00000186007	LEMD1	5.37	0.47	14.01	0.00	0.00	75.14
ENSG00000181577	C6orf223	5.36	4.49	14.02	0.00	0.00	75.26
ENSG00000108244	KRT23	5.33	3.67	5.86	0.00	0.00	9.46
ENSG00000015413	DPEP1	5.29	6.34	7.84	0.00	0.00	21.56
ENSG00000175832	ETV4	5.27	6.24	19.64	0.00	0.00	135.08
ENSG00000115507	OTX1	5.26	0.59	15.24	0.00	0.00	87.64
ENSG00000178773	CPNE7	5.04	3.88	10.47	0.00	0.00	42.15
ENSG00000185479	KRT6B	5.02	1.24	8.60	0.00	0.00	27.26
**LncRNA**							
ENSG00000214039	LINC02418	7.40	1.88	9.02	0.00	0.00	30.43
ENSG00000230316	FEZF1-AS1	6.45	−0.09	11.22	0.00	0.00	48.72
ENSG00000253929	CASC21	5.30	−0.60	13.65	0.00	0.00	71.58
ENSG00000281406	BLACAT1	5.15	1.48	15.00	0.00	0.00	85.11
ENSG00000229404	LINC00858	4.84	−0.95	10.67	0.00	0.00	43.88
ENSG00000275216	AL161431.1	4.74	0.98	8.30	0.00	0.00	25.05
ENSG00000259485	LINC02253	4.70	−0.54	9.85	0.00	0.00	36.98
ENSG00000236081	ELFN1-AS1	4.54	2.19	11.88	0.00	0.00	54.68
ENSG00000237686	AL109615.3	4.44	0.10	18.56	0.00	0.00	122.90
ENSG00000245694	CRNDE	4.17	1.18	12.86	0.00	0.00	63.93
ENSG00000254560	BBOX1-AS1	4.02	0.21	9.71	0.00	0.00	35.91
ENSG00000204876	AC021218.1	3.27	3.70	9.13	0.00	0.00	31.18
ENSG00000226476	LINC01748	3.02	−0.28	7.36	0.00	0.00	18.54
ENSG00000262188	LINC01978	3.01	0.53	9.07	0.00	0.00	30.80
ENSG00000253414	AC124067.2	2.98	0.78	9.77	0.00	0.00	36.38
ENSG00000214049	UCA1	2.94	2.88	5.57	0.00	0.00	7.82
ENSG00000265688	MAFG-AS1	2.94	2.20	17.51	0.00	0.00	111.62
ENSG00000230061	TRPM2-AS	2.81	1.05	5.77	0.00	0.00	9.02
ENSG00000253161	LINC01605	2.80	0.63	10.92	0.00	0.00	46.10
ENSG00000255026	AC136475.3	2.71	1.18	6.52	0.00	0.00	13.26
		**logFC**	**AveExpr**	**t**	***P*Value**	**FDR**	**B**
**miRNA**							
hsa-miR-374a-3p		7.69	9.55	15.73	0.00	0.00	90.87
hsa-miR-135b-5p		6.45	5.85	8.48	0.00	0.00	26.20
hsa-miR-21-5p		6.32	17.44	27.96	0.00	0.00	219.16
hsa-miR-19b-3p		6.31	7.48	13.27	0.00	0.00	66.88
hsa-miR-142-3p		6.20	10.81	11.47	0.00	0.00	50.44
hsa-miR-19a-3p		6.16	5.36	10.03	0.00	0.00	38.20
hsa-miR-424-5p		6.07	6.75	12.54	0.00	0.00	59.99
hsa-miR-142-5p		6.04	6.12	12.36	0.00	0.00	58.34
hsa-miR-542-3p		5.74	7.00	14.22	0.00	0.00	75.91
hsa-miR-577		5.59	5.62	5.91	0.00	0.00	9.83
hsa-miR-29b-3p		5.40	9.40	13.88	0.00	0.00	72.73
hsa-miR-126-5p		5.24	7.06	13.04	0.00	0.00	64.66
hsa-miR-32-5p		5.23	4.63	14.04	0.00	0.00	74.18
hsa-miR-33a-5p		5.13	5.14	7.48	0.00	0.00	19.30
hsa-miR-582-3p		5.08	8.28	11.93	0.00	0.00	54.45
hsa-miR-203b-3p		5.05	7.07	7.03	0.00	0.00	16.42
hsa-miR-101-3p		5.01	12.47	21.16	0.00	0.00	148.02
hsa-miR-18a-5p		4.99	4.50	9.33	0.00	0.00	32.60
hsa-miR-429		4.93	8.33	11.45	0.00	0.00	50.22
hsa-miR-374a-5p		4.91	4.77	12.57	0.00	0.00	60.33

**Table 3:** Top 20 downregulated mRNAs, lncRNAs, and miRNAs

	**symbol**	**logFC**	**AveExpr**	**t**	***P*Value**	**FDR**	**B**
**mRNA**							
ENSG00000104267	CA2	−5.61	5.14	−18.17	0.00	0.00	118.92
ENSG00000248144	ADH1C	−5.61	3.53	−17.14	0.00	0.00	107.73
ENSG00000007306	CEACAM7	−5.62	6.08	−11.41	0.00	0.00	50.10
ENSG00000269404	SPIB	−5.71	0.57	−34.77	0.00	0.00	298.79
ENSG00000168079	SCARA5	−6.00	1.08	−30.10	0.00	0.00	250.25
ENSG00000109182	CWH43	−6.01	−0.28	−23.36	0.00	0.00	176.45
ENSG00000080493	SLC4A4	−6.01	2.61	−23.50	0.00	0.00	178.10
ENSG00000016490	CLCA1	−6.37	4.95	−9.29	0.00	0.00	32.04
ENSG00000142959	BEST4	−6.53	0.29	−45.79	0.00	0.00	402.65
ENSG00000196616	ADH1B	−6.69	0.74	−26.47	0.00	0.00	210.91
ENSG00000091138	SLC26A3	−6.79	5.53	−11.68	0.00	0.00	52.58
ENSG00000197273	GUCA2A	−6.81	2.83	−24.00	0.00	0.00	183.57
ENSG00000167080	B4GALNT2	−6.82	0.89	−21.91	0.00	0.00	160.29
ENSG00000204936	CD177	−7.45	1.83	−26.62	0.00	0.00	212.57
ENSG00000100604	CHGA	−7.45	1.25	−25.15	0.00	0.00	196.43
ENSG00000167434	CA4	−7.77	1.59	−23.02	0.00	0.00	172.71
ENSG00000071203	MS4A12	−7.93	0.78	−24.35	0.00	0.00	187.48
ENSG00000174992	ZG16	−8.12	2.90	−18.31	0.00	0.00	120.48
ENSG00000016602	CLCA4	−8.41	2.08	−22.49	0.00	0.00	166.82
ENSG00000103375	AQP8	−9.02	0.89	−27.39	0.00	0.00	220.97
**LncRNA**							
ENSG00000186594	MIR22HG	−1.92	3.37	−17.54	0.00	0.00	112.01
ENSG00000227258	SMIM2-AS1	−1.92	1.43	−9.98	0.00	0.00	37.78
ENSG00000167912	AC090152.1	−1.96	0.17	−10.72	0.00	0.00	44.25
ENSG00000224259	LINC01133	−1.97	3.44	−12.97	0.00	0.00	64.65
ENSG00000167117	LINC00483	−2.01	3.21	−11.42	0.00	0.00	50.12
ENSG00000225953	SATB2-AS1	−2.02	2.74	−8.48	0.00	0.00	25.84
ENSG00000266036	AC016888.1	−2.12	0.23	−13.73	0.00	0.00	72.38
ENSG00000229155	LINC02038	−2.14	0.02	−16.45	0.00	0.00	100.45
ENSG00000268388	FENDRR	−2.17	3.13	−12.79	0.00	0.00	62.96
ENSG00000237070	AC005550.3	−2.19	0.16	−9.16	0.00	0.00	31.33
ENSG00000276855	AC015922.4	−2.22	0.04	−12.81	0.00	0.00	63.38
ENSG00000258837	AL133370.1	−2.37	0.49	−7.89	0.00	0.00	21.86
ENSG00000198788	MUC2	−2.40	8.10	−4.33	0.00	0.00	1.46
ENSG00000229619	MBNL1-AS1	−2.41	1.31	−19.67	0.00	0.00	135.40
ENSG00000259342	AC025580.1	−2.46	1.56	−12.25	0.00	0.00	57.93
ENSG00000225335	AC016027.1	−2.50	0.61	−26.88	0.00	0.00	215.20
ENSG00000188242	PP7080	−2.75	4.85	−14.61	0.00	0.00	81.01
ENSG00000224189	HAGLR	−2.91	2.84	−15.85	0.00	0.00	93.94
ENSG00000226777	FAM30A	−3.26	−0.28	−13.10	0.00	0.00	66.15
ENSG00000256643	LINC02441	−3.29	−0.05	−13.32	0.00	0.00	68.27
		**logFC**	**AveExpr**	**t**	**PValue**	**FDR**	**B**
**miRNA**							
hsa-miR-378a-5p		−4.20	6.76	−15.80	0.00	0.00	91.92
hsa-miR-1180-3p		−4.23	3.03	−11.14	0.00	0.00	47.49
hsa-miR-150-3p		−4.27	0.56	−9.24	0.00	0.00	31.83
hsa-miR-671-3p		−4.28	1.31	−16.20	0.00	0.00	95.92
hsa-let-7d-3p		−4.34	8.50	−18.59	0.00	0.00	120.89
hsa-miR-125a-5p		−4.39	7.80	−16.53	0.00	0.00	99.38
hsa-miR-1976		−4.44	2.84	−18.10	0.00	0.00	115.64
hsa-miR-1306-5p		−4.46	2.46	−17.48	0.00	0.00	109.21
hsa-miR-149-5p		−4.49	3.27	−13.05	0.00	0.00	64.85
hsa-miR-766-3p		−4.50	2.50	−18.10	0.00	0.00	115.63
hsa-miR-194-3p		−4.51	7.68	−13.59	0.00	0.00	70.05
hsa-miR-133a-3p		−4.59	3.71	−8.32	0.00	0.00	24.94
hsa-miR-197-3p		−4.97	7.87	−22.64	0.00	0.00	163.76
hsa-miR-642a-5p		−5.07	1.39	−11.47	0.00	0.00	50.39
hsa-miR-6511b-3p		−5.37	0.31	−17.29	0.00	0.00	107.15
hsa-miR-139-5p		−5.38	4.32	−17.29	0.00	0.00	107.26
hsa-miR-328-3p		−5.63	3.77	−21.46	0.00	0.00	151.16
hsa-miR-129-5p		−5.76	0.92	−13.35	0.00	0.00	67.73
hsa-miR-139-3p		−6.10	1.99	−14.44	0.00	0.00	78.35
hsa-miR-486-5p		−6.12	5.62	−14.35	0.00	0.00	77.43

### GO enrichment and KEGG pathway analysis

**Fig. 3:**
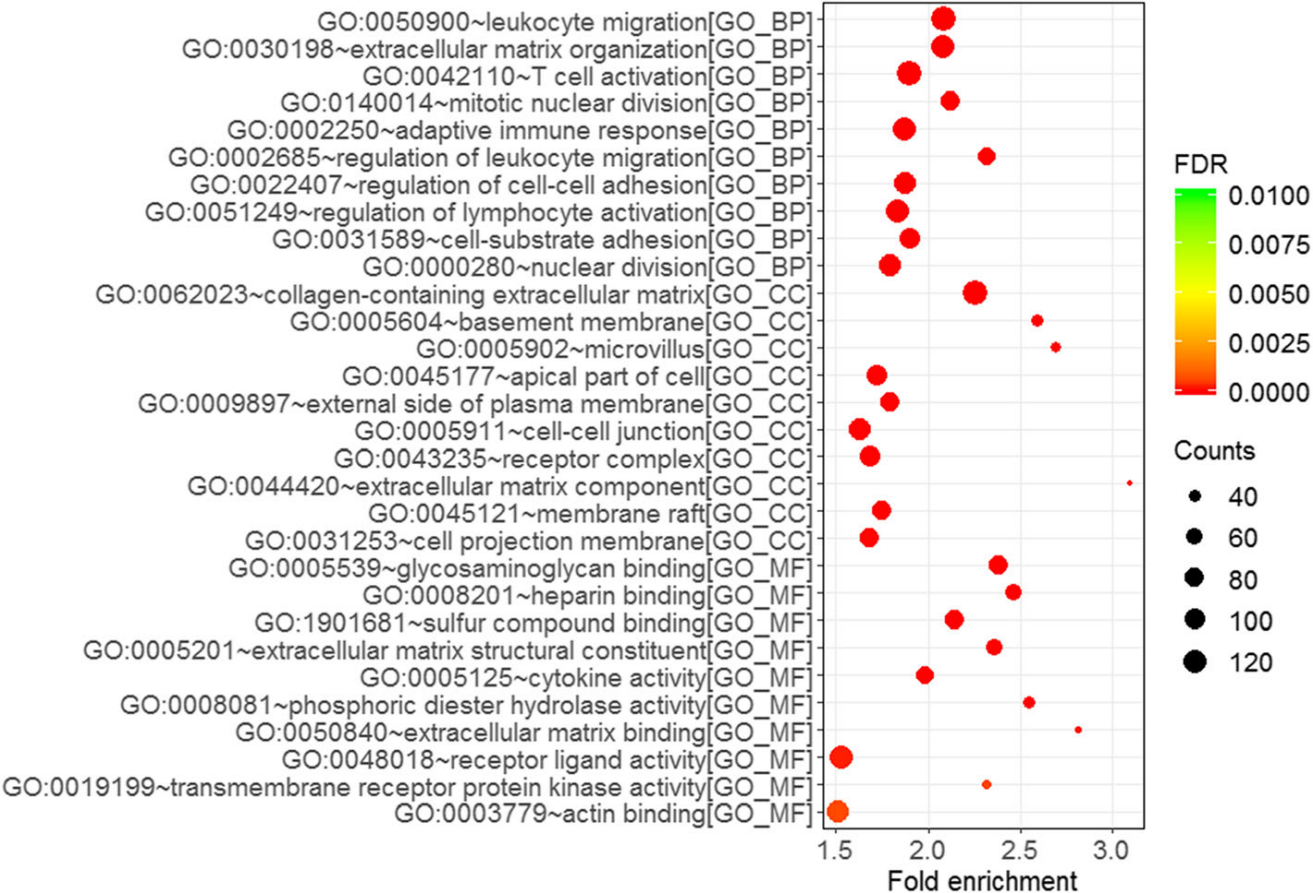
GO enrichment analysis of the differentially expressed mRNAs in COAD (Top 10 GO enrichment are presented)

**Fig. 4:**
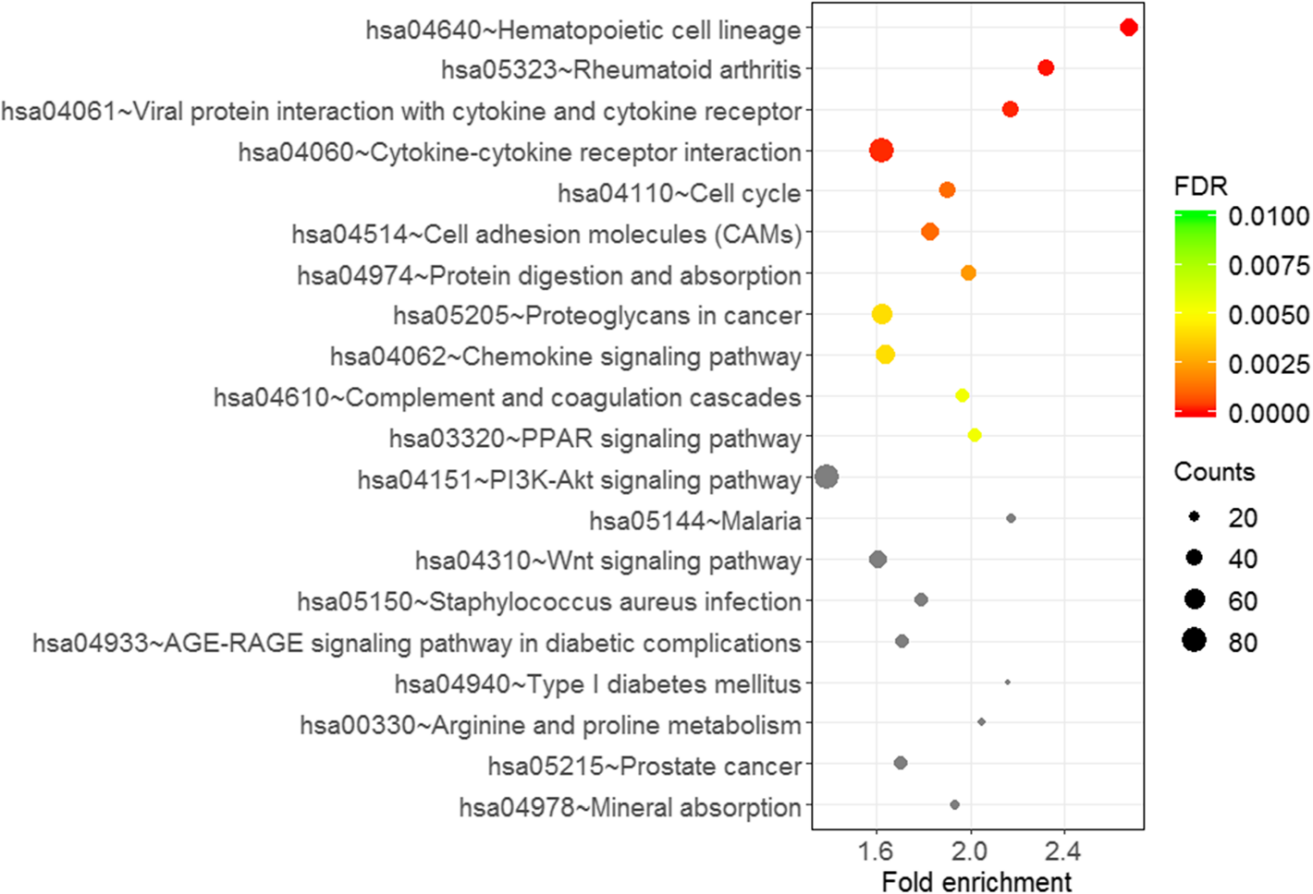
KEGG signaling pathway analysis of the differentially expressed mRNAs in COAD (Top 20 KEGG terms are presented)

GO enrichment analysis demonstrated that the differentially expressed mRNAs were enriched in different biological processes such as leukocyte migration, extracellular matrix organization, T cell activation, mitotic nuclear division, and adaptive immune response. Furthermore, GO analysis in cellular component revealed that the differentially expressed mRNAs predominantly contributed to collagen-containing extracellular matrix, basement membrane, microvillus, apical part of cell, and external side of plasma membrane. GO molecular function domain indicated that the genes were mainly enriched in glycosaminoglycan binding, heparin binding, sulfur compound binding, extracellular matrix structural constituent, and cytokine activity. GO outputs are presented in Fig. 3. In addition, KEGG pathway analysis indicated that the differentially expressed genes in the COAD patients remarkably participated in pathways involving in cancer, cell cycle, PPAR signaling pathway, PI3K-Akt signaling pathway, Wnt signaling pathway, and p53 signaling pathway (Fig. 4).

### PPI network construction

**Fig. 5:**
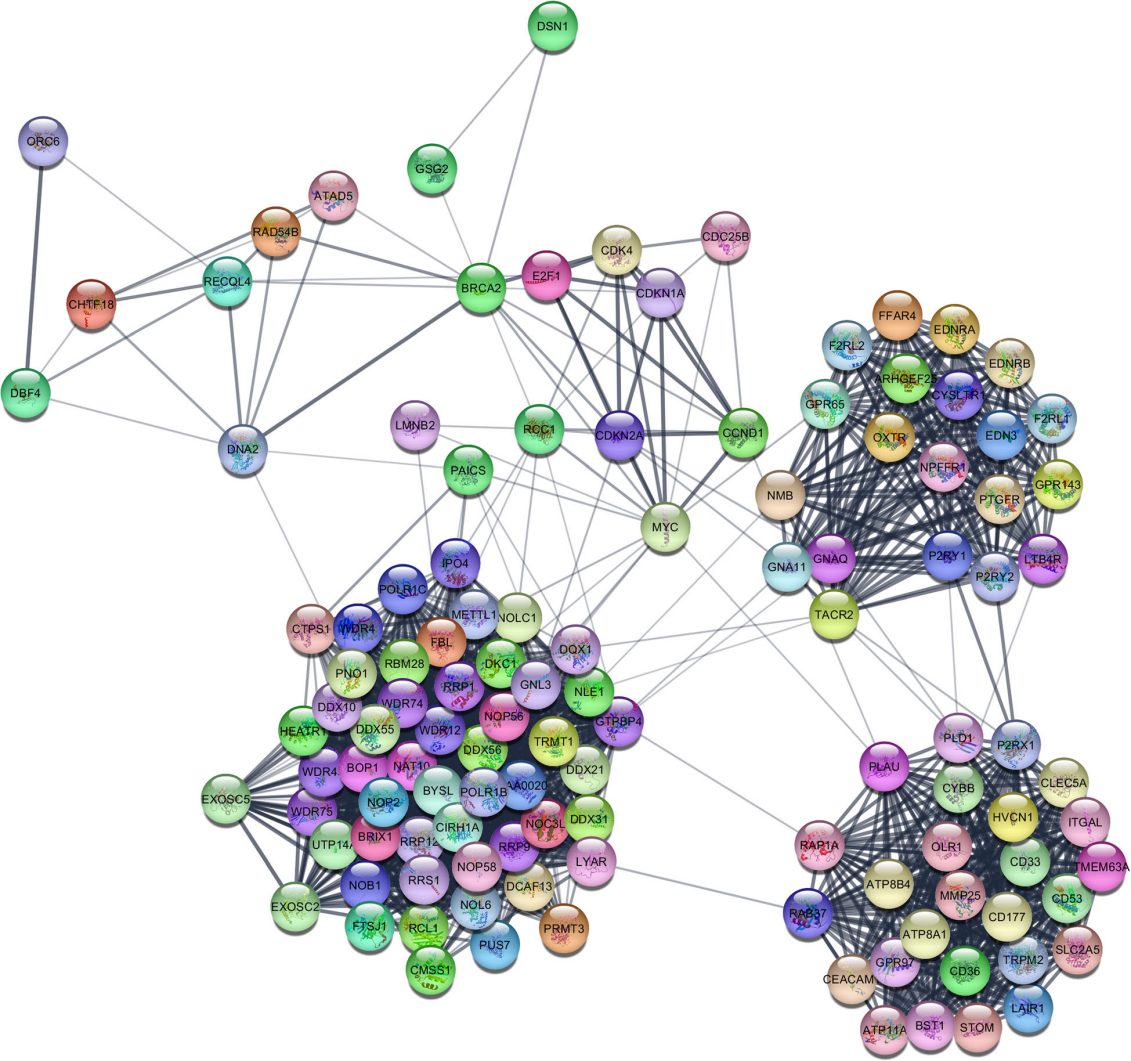
Protein-protein interaction (PPI) network of the differentially mRNAs in COAD (score > 0.4) with Node:118, eadge:1745, MCADE score: 29.82

The PPI network was constructed based on the STRING database to better understand the roles of the differentially expressed mRNAs. The data demonstrated that CDKN2A, CCND1, MYC, E2F, CDK4, BRCA2, CDC25B, and CDKN1A were the protein-protein interaction (PPI) critical hubs (Fig. 5).

### Kaplan-Meier survival analysis of differentially expressed genes

Kaplan-Meier survival analysis was used to indicate the association of differentially expressed mRNAs, lncRNAs, miRNA, and prognosis of COAD patients. The data showed that 215 mRNAs, 14 lncRNAs, and 39 miRNAs were associated with overall survival time in the patients. The top 10 hits of each group are presented in Table 4.

**Table 4:** Top 10 mRNAs, lncRNAs, and miRNAs that were associated with overall survival

	symbol	HR	lower95	upper95	*p *Value
mRNA					
ENSG00000204314	PRRT1	2.11	1.43	3.12	0.00
ENSG00000179528	LBX2	2.09	1.42	3.08	0.00
ENSG00000108852	MPP2	2.08	1.41	3.07	0.00
ENSG00000225968	ELFN1	1.99	1.35	2.93	0.00
ENSG00000258839	MC1R	1.94	1.32	2.86	0.00
ENSG00000187730	GABRD	1.94	1.31	2.86	0.00
ENSG00000163083	INHBB	1.92	1.30	2.83	0.00
ENSG00000204389	HSPA1A	1.91	1.29	2.81	0.00
ENSG00000124191	TOX2	1.88	1.28	2.77	0.00
ENSG00000198467	TPM2	1.83	1.24	2.70	0.00
LncRNA					
ENSG00000262251	AC087388.1	1.86	1.26	2.74	0.00
ENSG00000226419	SLC16A1-AS1	1.83	1.24	2.69	0.00
ENSG00000236081	ELFN1-AS1	1.74	1.18	2.57	0.01
ENSG00000267523	AC008735.2	1.66	1.12	2.45	0.01
ENSG00000226332	AL354836.1	1.66	1.12	2.44	0.01
ENSG00000273142	AC073335.2	1.51	1.02	2.22	0.04
ENSG00000278709	NKILA	1.51	1.02	2.22	0.04
ENSG00000254815	AP006284.1	1.50	1.02	2.22	0.04
ENSG00000234432	AC092171.3	1.49	1.01	2.19	0.05
ENSG00000228109	MELTF-AS1	1.48	1.00	2.18	0.05
miRNA					
hsa-miR-130a-3p		1.84	1.24	2.72	0.00
hsa-miR-210-3p		1.79	1.21	2.65	0.00
hsa-miR-193a-3p		1.78	1.21	2.63	0.00
hsa-miR-887-3p		1.76	1.19	2.59	0.01
hsa-miR-34a-5p		1.69	1.14	2.50	0.01
hsa-miR-34c-5p		1.66	1.12	2.45	0.01
hsa-miR-26b-5p		1.65	1.11	2.43	0.01
hsa-miR-193b-5p		1.63	1.10	2.40	0.02
hsa-miR-328-3p		1.62	1.10	2.40	0.02
hsa-miR-1271-5p		1.61	1.09	2.38	0.02

### Diagnostic analysis of differentially expressed lncRNAs

AUC analysis was conducted to demonstrate the diagnostic value of each lncRNAs in the COAD samples. All 205 differentially expressed lncRNAs indicated significant diagnostic values. The top 50 hits of the lncRNAs are summarized in Table 5.

**Table 5:** Top 50 lncRNAs that had significant diagnostic value

LncRNA	symbol	Area	SE	*p*-value	Lower Bound	Upper Bound	Expression
ENSG00000249859	PVT1	1.00	0.00	0.00	1.00	1.00	High
ENSG00000265688	MAFG-AS1	1.00	0.00	0.00	0.99	1.00	High
ENSG00000237686	AL109615.3	0.99	0.00	0.00	0.98	1.00	High
ENSG00000232956	SNHG15	0.98	0.01	0.00	0.97	1.00	High
ENSG00000281406	BLACAT1	0.98	0.01	0.00	0.97	0.99	High
ENSG00000236081	ELFN1-AS1	0.98	0.01	0.00	0.97	0.99	High
ENSG00000245694	CRNDE	0.98	0.01	0.00	0.97	0.99	High
ENSG00000163597	SNHG16	0.98	0.01	0.00	0.96	0.99	High
ENSG00000186594	MIR22HG	0.97	0.01	0.00	0.01	0.04	Low
ENSG00000225335	AC016027.1	0.97	0.02	0.00	0.00	0.06	Low
ENSG00000253929	CASC21	0.97	0.01	0.00	0.96	0.98	High
ENSG00000268388	FENDRR	0.97	0.01	0.00	0.02	0.05	Low
ENSG00000255717	SNHG1	0.97	0.01	0.00	0.95	0.98	High
ENSG00000203497	PDCD4-AS1	0.97	0.02	0.00	0.00	0.07	Low
ENSG00000256643	LINC02441	0.96	0.02	0.00	0.01	0.07	Low
ENSG00000280798	LINC00294	0.96	0.01	0.00	0.01	0.06	Low
ENSG00000270820	AC016727.1	0.96	0.01	0.00	0.02	0.06	Low
ENSG00000272686	AC006333.2	0.96	0.01	0.00	0.02	0.06	Low
ENSG00000230316	FEZF1-AS1	0.96	0.01	0.00	0.94	0.98	High
ENSG00000262001	DLGAP1-AS2	0.96	0.01	0.00	0.94	0.98	High
ENSG00000177410	ZFAS1	0.96	0.01	0.00	0.94	0.98	High
ENSG00000224189	HAGLR	0.96	0.01	0.00	0.02	0.06	Low
ENSG00000253161	LINC01605	0.96	0.01	0.00	0.94	0.98	High
ENSG00000270959	LPP-AS2	0.96	0.01	0.00	0.03	0.06	Low
ENSG00000196756	SNHG17	0.96	0.01	0.00	0.93	0.98	High
ENSG00000272106	AL691432.2	0.96	0.01	0.00	0.02	0.07	Low
ENSG00000228109	MELTF-AS1	0.95	0.01	0.00	0.93	0.98	High
ENSG00000261373	VPS9D1-AS1	0.95	0.01	0.00	0.94	0.97	High
ENSG00000229619	MBNL1-AS1	0.95	0.01	0.00	0.03	0.07	Low
ENSG00000234753	FOXP4-AS1	0.95	0.01	0.00	0.93	0.97	High
ENSG00000281376	ABALON	0.95	0.01	0.00	0.93	0.97	High
ENSG00000276855	AC015922.4	0.95	0.01	0.00	0.03	0.07	Low
ENSG00000229155	LINC02038	0.95	0.02	0.00	0.01	0.10	Low
ENSG00000226380	AC016831.1	0.95	0.01	0.00	0.93	0.97	High
ENSG00000253414	AC124067.2	0.95	0.01	0.00	0.93	0.97	High
ENSG00000266680	AL135905.2	0.95	0.02	0.00	0.02	0.09	Low
ENSG00000256940	AP001453.2	0.94	0.02	0.00	0.91	0.97	High
ENSG00000243479	MNX1-AS1	0.94	0.01	0.00	0.91	0.97	High
ENSG00000245910	SNHG6	0.94	0.01	0.00	0.91	0.96	High
ENSG00000272502	AC104958.2	0.94	0.01	0.00	0.92	0.96	High
ENSG00000172965	MIR4435-2HG	0.94	0.02	0.00	0.89	0.98	High
ENSG00000236144	TMEM147-AS1	0.93	0.01	0.00	0.91	0.96	High
ENSG00000214039	LINC02418	0.93	0.01	0.00	0.91	0.96	High
ENSG00000272462	U91328.2	0.93	0.01	0.00	0.04	0.09	Low
ENSG00000280206	AC026401.3	0.93	0.02	0.00	0.90	0.97	High
ENSG00000205664	BX890604.1	0.93	0.02	0.00	0.91	0.96	High
ENSG00000262585	LINC01979	0.93	0.01	0.00	0.91	0.95	High
ENSG00000262188	LINC01978	0.93	0.01	0.00	0.90	0.96	High
ENSG00000166770	ZNF667-AS1	0.93	0.01	0.00	0.05	0.10	Low
ENSG00000232442	MHENCR	0.93	0.01	0.00	0.90	0.95	High

### Novel lncRNA biomarkers

After merging the overall survival, and the diagnostic value data, we found that 14 lncRNAs had high ranks in prognostic and diagnostic areas which can be considered as COAD biomarkers. The data are presented in Table 6.

**Table 6:** the lncRNAs as diagnostic and prognostic biomarkers in COAD

**LncRNA**	**symbol**	**Area**	SE	***p*-value**	**Expression**	**HR**	***p*Value**
ENSG00000262251	AC087388.1	0.89	0.02	0.00	High	1.86	0.00
ENSG00000226419	SLC16A1-AS1	0.88	0.02	0.00	High	1.83	0.00
ENSG00000236081	ELFN1-AS1	0.98	0.01	0.00	High	1.74	0.01
ENSG00000254290	AC124067.4	0.79	0.02	0.00	High	0.58	0.01
ENSG00000265415	AC099850.3	0.89	0.02	0.00	High	0.60	0.01
ENSG00000267523	AC008735.2	0.78	0.03	0.00	High	1.66	0.01
ENSG00000226332	AL354836.1	0.86	0.02	0.00	High	1.66	0.01
ENSG00000268388	FENDRR	0.97	0.01	0.00	Low	0.60	0.01
ENSG00000260920	AL031985.3	0.87	0.02	0.00	High	0.64	0.03
ENSG00000278709	NKILA	0.79	0.02	0.00	High	1.51	0.04
ENSG00000254815	AP006284.1	0.76	0.03	0.00	High	1.50	0.04
ENSG00000273142	AC073335.2	0.83	0.02	0.00	High	1.51	0.04
ENSG00000234432	AC092171.3	0.88	0.02	0.00	High	1.49	0.05
ENSG00000228109	MELTF-AS1	0.95	0.01	0.00	High	1.48	0.05

**Fig. 6:**
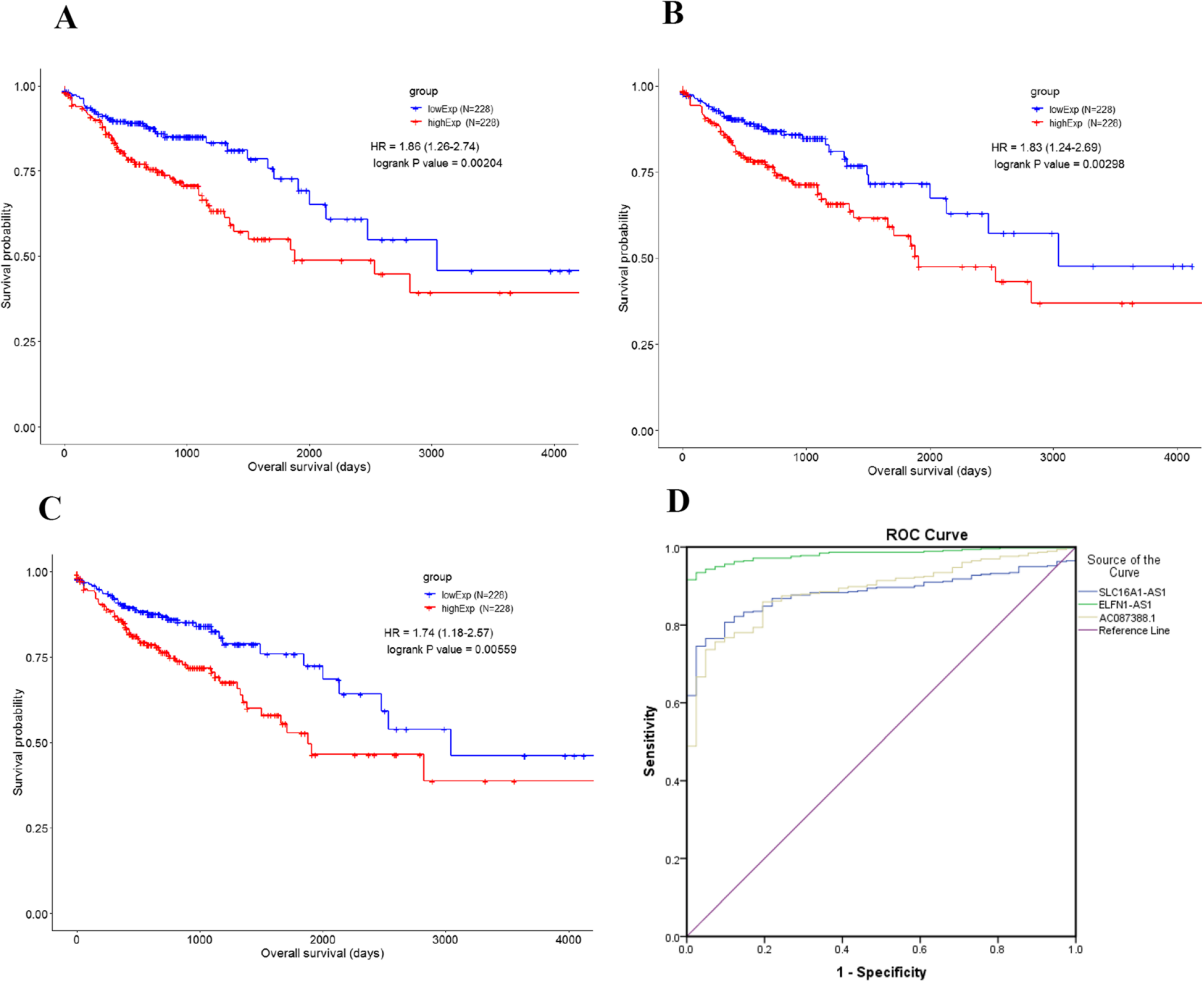
Kaplan-Meier and ROC curve analysis of the AC087388.1, SLC16A1-AS1, and ELFN1-AS1). **a** Kaplan-Meier curve of AC087388.1. **b** Kaplan-Meier curve of SLC16A1-AS. **c** Kaplan-Meier curve of ELFN1-AS1. **d** ROC curve of the lncRNAs

Kaplan-Meier and ROC curve analysis were conducted for the top three lncRNAs (AC087388.1, SLC16A1-AS1, and ELFN1-AS1) from aforementioned analysis shown in Fig. 6. Moreover, univariate and multivariate analysis were conducted to demonstrate the power of the lncRNAs and to diminish the covariate effects. Univariate and multivariate analysis are summarized in Table 7.

**Table 7:** Univariate and multivariate survival analyses of AC087388.1, SLC16A1-AS1, and ELFN1-AS1

					**AC087388**				**SLC16A1-AS1**				**ELFN1-AS1**			
	**Univariate analysis**				**Multivariate analysis**				**Multivariate analysis**				**Multivariate analysis**			
	**HR**	**95% CI**		***P*value**	**HR**	**95% CI**		***P*value**	**HR**	**95% CI**		***P*value**	**HR**	**95% CI**		***P*value**
**ENSG00000262251**	1.45	1.14	1.85	0.00	1.53	1.01	2.31	0.04								
**ENSG00000226419**	1.22	1.01	1.47	0.04					1.95	1.27	3.00	0.00				
**ENSG00000236081**	1.15	1.01	1.31	0.04									1.86	1.23	2.81	0.00
**Stage1&2/3&4**	2.73	1.80	4.15	0.00	1.68	0.96	2.91	0.07	1.58	0.90	2.76	0.11	1.64	0.95	2.85	0.08
**Pathologic_T (T1&2/T3&4)**	2.95	1.37	6.37	0.01	2.20	0.86	5.63	0.10	2.42	0.95	6.19	0.06	2.38	0.93	6.07	0.07
**pathologic_M (M0/Mx)**	3.12	2.08	4.68	0.00	2.15	1.36	3.40	0.00	2.26	1.43	3.57	0.00	2.34	1.49	3.70	0.00
**Pathologic_N (N0&1/N2)**	3.28	2.19	4.93	0.00	1.71	1.03	2.82	0.04	1.65	1.00	2.73	0.05	1.68	1.01	2.79	0.04
**Sex (Female/Male)**	1.09	0.74	1.61	0.68	0.93	0.62	1.41	0.73	0.92	0.61	1.39	0.69	0.90	0.59	1.36	0.61
**Age (≤65/> 65)**	1.76	1.18	2.63	0.01	2.25	1.46	3.48	0.00	2.49	1.61	3.86	0.00	2.29	1.49	3.54	0.00

### LncRNA-miRNA-mRNA ceRNA network construction

**Fig. 7:**
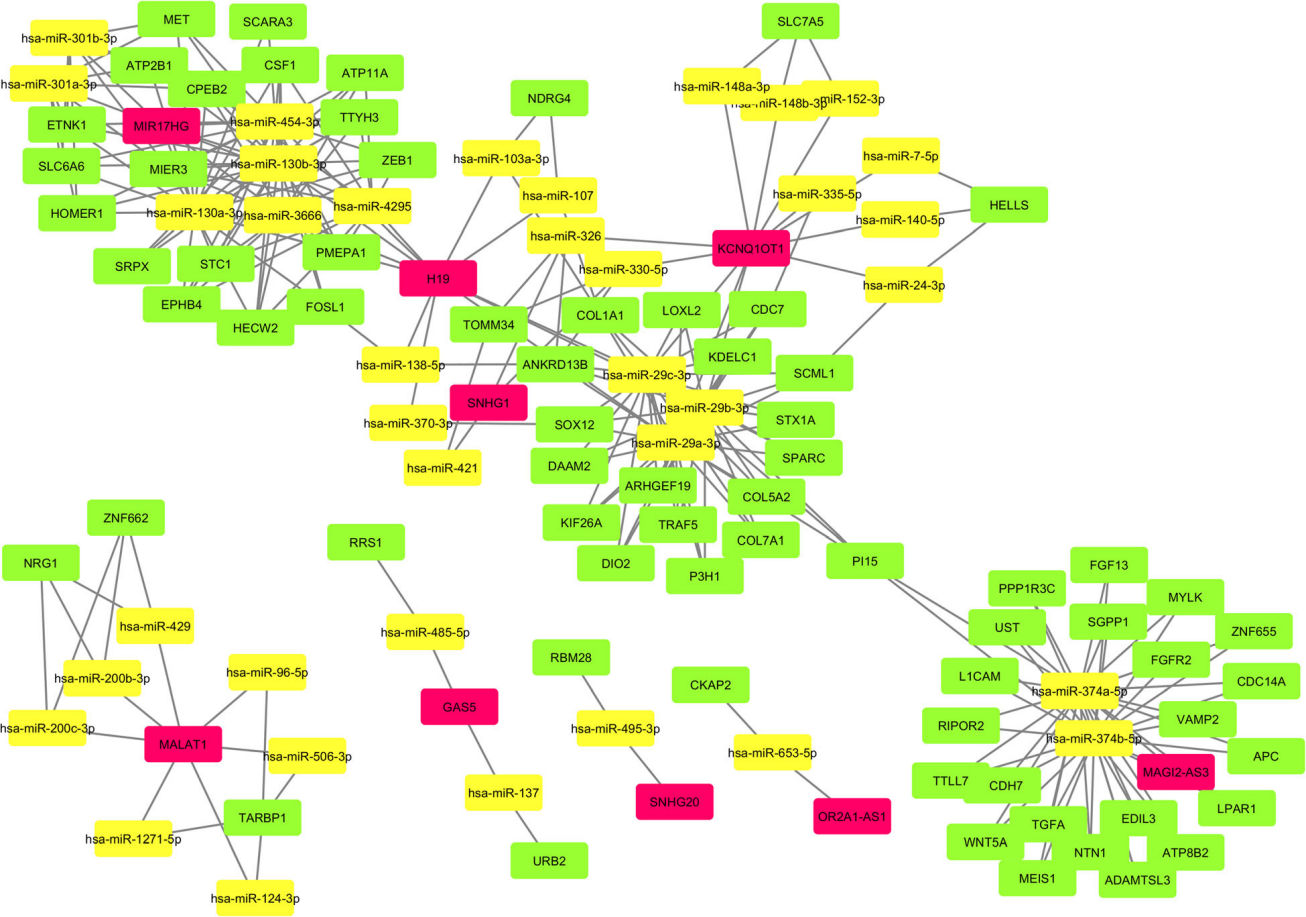
LncRNA-miRNA-mRNA ceRNA network construction of the differentially expressed genes in COAD (Red: LncRNA, Yellow: miRNA, and Green: mRNA)

According to ceRNA hypothesis, which implicates that lncRNAs regulate mRNA expression level by competing the shared miRNAs in cells, a ceRNA network was built based on lncRNAs, mRNAs, and miRNAs expression in the samples based on starbase online tool in R software. The nodes and edges were drawn by Cytoscape 3.7.2. The ceRNA network data demonstrated important lncRNAs including *MIR17HG, H19, SNHG1, KCNQ1OT1, MALAT1, GAS5, SNHG20, OR2A1-AS1,* and *MAGI2-AS3,* which have implied in the development of COAD (Fig. 7).

## Discussion

LncRNAs regulate critical and canonical biological functions in different types of normal human cells and in a variety of tumor cells [20]. An escalating number of investigations have reported the function of lncRNAs in tumor proliferation, cell invasion and migration, chemotherapy resistance, and stemness capability in tumorigenesis and progression of COAD [21–23]. However, the exact underlying mechanisms of lncRNAs in progression of COAD are still unclear. So far, several different biological regulatory functions have been proposed for lncRNAs. Some previous studies have demonstrated that lncRNAs regulate mRNA expression via binding and sponging miRNA known as competing endogenous RNA theory, which generates a new aspect in the lncRNA regulatory mechanism [24, 25]. To the best of our knowledge, only a few investigations have displayed ceRNA networks between lncRNAs and miRNAs in COAD. Thus, a clear image of lncRNAs-miRNAs links still remains uncharacterized. In the current study, we studied the differentially expressed genes including lncRNAs, miRNAs, and mRNAs in the COAD patients based on TCGA database. Gene set enrichment by GO and KEGG signaling pathway identified the differentially expressed genes which were significantly enriched in cell proliferation, differentiation, protein phosphorylation, and signaling pathways. Furthermore, KEGG signaling pathway analysis demonstrated several canonical signaling pathways including Wnt, PI3K/Akt and PPAR signaling pathways that have been shown to contribute in tumor progression [26, 27]. A mounting of evidence has emphasized on Wnt/ β-catenin signaling pathway, promoting tumor growth, invasion and metastasis, and chemoresistance in COAD [28, 29]. For instance, it has been demonstrated that lncRNA *H19* overexpression induces the EMT of colorectal cancer (CRC) cells by sponging *miR-29b-3p* to directly upregulate *PGRN* and activate Wnt axis [30]. Moreover, the up-regulation of lncRNA *colorectal cancer-associated lncRNA* (*CCAL*) promotes CRC progression through suppressing the *activator protein 2α (AP-2α*) to initiate Wnt/β-catenin signaling pathway [31]. In the present study, the KEGG analysis indicated that the peroxisome proliferator-activated receptor (PPAR) pathway contributes in Wnt signaling. It has been shown that the PPAR signaling pathway reduces cell proliferation and inhibits tumorigenesis in different types of cancers. Down-regulation of *PPAR-α* has been correlated with poor clinicopathological features of CRC that was remarkably higher in well to moderately differentiated adenocarcinoma than in mucinous adenocarcinoma [32]. In addition, lncRNA *TINCR* modulates PPAR signaling pathway through binding to *miR-107* to up-regulate *CD36* in CRC [33]. Recently, the PPAR aberration expression and its prime roles in gastrointestinal tract has been extensively reviewed [34].

It has been shown that PI3K/Akt signaling pathway had prominent roles in carcinogenesis of a variety of cancers particularly COAD. LncRNA *AB073614* can take under control CRC growth and invasion by PI3K/Akt signaling pathway [35]. In addition, lncRNA *SNHG7* elevated *GALNT7* level and induced PI3K/Akt/mTOR pathway by sponging *miR-34a* in CRC cells [36]. Our ceRNA network data demonstrated important lncRNAs including *MIR17HG, H19, SNHG1, KCNQ1OT1, MALAT1, GAS5, SNHG20, OR2A1-AS1,* and *MAGI2-AS3* which previously have been highlighted in the development of COAD. LncRNA *MAGI2-AS3* have been discovered to play a crucial role as a tumor suppressor in breast cancer by targeting *Fas/FasL* in tumor cells [37]. Moreover, *MAGI2-AS3* hampers hepatocellular carcinoma cell growth and its invasion through sponging *miR-374b-5p* to up-regulate *SMG1* axis [38]. On the other hand, overexpression of *MAGI2-AS3* has been explained to promote tumor progression by absorbing *miR-141/200a* and consequently, up-regulating *ZEB1* which is an EMT promoting transcription factor, in gastric cancer cells [39]. *MAGI2-AS3* up-regulation has also been shown to induce CRC proliferation and migration by modulating *miR-3163* through upregulating *TMEM106B* [40].

LncRNA *SNHG1* is a prominent lncRNA that is involved in a variety of cancers. *SNHG1* expression is associated with unfavorable overall survival and tumor recurrence in patients with COAD. Moreover, *SNHG1* promote cell growth and cell migration via upregulating *EZH2* and *miR-154a-5p* in COAD [41]. LncRNA *KCNQ1OT1* can promote EMT by decreasing *miRNA-217* expression to upregulate *ZEB1* axis in COAD [42]. Furthermore, *KCNQ1OT1* has been demonstrated to promote chemoresistance of oxaliplatin by iR-34a/ATG4B pathway and it is associated with poor prognosis in COAD [43]. A previous study showed that lncRNA *MALAT1* was remarkably upregulated in COAD cells. *MALAT1* can promote metastasis of COAD via *RUNX2* as a survival factor in tumor cells [44]. *MALAT1* evokes EMT and angiogenesis via sponging *miR-1265p* to upregulate *VEGFA*, *SLUG*, and *TWIST* [45]. Several investigations demonstrated that lncRNA *GAS5* can act as a tumor suppressor gene by different actions. It has been illustrated that *GAS5* inhibited angiogenesis and metastasis via regulating Wnt signaling pathway in COAD cells [46]. Finally, lncRNA SNHG20 has been reported overexpressed prominently in CRC tissues in comparison to normal ones. Overexpression of SNHG20 was correlated with poor prognosis in the patients [47]. Although, there are several similar studies, the novelties of the current study include; an extensive exploration of lncRNA, mRNA and miRNA signatures, revealing the diagnostic and prognostic value of lncRNA, and constructing a COAD lncRNA-miRNA-mRNA ceRNA network.

Hence, our data elucidated that, the suggested lncRNAs can be considered as potential promising biomarkers, which could drive tumorigenesis through hijacking canonical signaling pathways in COAD.

## Conclusions

Our data highlighted the importance of lncRNA regulatory networks that might provide a promising therapeutic approach for clinical application by considering lncRNA hubs as potential efficient biomarkers.

## Data Availability

The miRNA-Seq and RNA-Seq genotype data analyzed in the current study are available through the open access datasets retrieved from TCGA [Project ID: TCGA-COAD, Project Name: Colon Adenocarcinoma (dbGaP Study Accession: phs000178)].

## Abbreviations

COAD: Colon adenocarcinoma
GI: Gastrointestinal
LncRNA: Long non-coding RNA
ORF: Open-reading-frame
ceRNA: Competing endogenous RNA
EMT: Epithelial-mesenchymal transition
DEmRNAs: Differentially expressed mRNAs
KEGG: Kyoto Encyclopedia of Genes and Genomes
CAMs: Cell adhesion molecules
CRC: Colorectal cancer
CCAL: Colorectal cancer-associated lncRNA
AP-2α: Activator protein 2α
PPAR: Peroxisome proliferator-activated receptor
